# Клинико-демографический анализ структуры телемедицинских консультаций «врач-пациент» в ФГБУ «НМИЦ эндокринологии» Минздрава России

**DOI:** 10.14341/probl13088

**Published:** 2022-04-01

**Authors:** А. М. Горбачева, О. В. Логвинова, Н. Г. Мокрышева

**Affiliations:** Национальный медицинский исследовательский центр эндокринологии; Национальный медицинский исследовательский центр эндокринологии; Национальный медицинский исследовательский центр эндокринологии

**Keywords:** телемедицина, эндокринология, организация здравоохранения

## Abstract

**ОБОСНОВАНИЕ:**

ОБОСНОВАНИЕ. Пандемия COVID-19 ускорила развитие телемедицинских технологий, на сегодняшний день имеются данные об успешном применении телемедицины в различных сферах здравоохранения, в частности, в эндокринологии. В то же время, информации, которая бы позволила эффективно интегрировать телемедицину в практику лечения пациентов с различными эндокринопатиями, недостаточно.

**ЦЕЛЬ:**

ЦЕЛЬ. Целью данной работы является клинико-демографическая оценка структуры телемедицинских консультаций (ТМК), проведенных в ФГБУ «НМИЦ эндокринологии» в 2020-2021 годах.

**МАТЕРИАЛЫ И МЕТОДЫ:**

МАТЕРИАЛЫ И МЕТОДЫ. Было проведено одномоментное одноцентровое ретроспективное исследование. В исследование были включены все пациенты, получившие хотя бы одну телемедицинскую консультацию в ФГБУ «НМИЦ эндокринологии» Минздрава России в 2020-2021гг. Анализировалась клинико-демографическая информация (пол, возраст пациентов, регион проживания, код заболевания по МКБ-10). Все пациенты подписали добровольное информированное согласие на проведение телемедицинской консультации. Обработка полученных данных проводилась при помощи пакета программ Microsoft Office версии 2013 года.

**РЕЗУЛЬТАТЫ:**

РЕЗУЛЬТАТЫ. В 2020г было проведено 1548 ТМК, в 2021г – 4180 ТМК. Среди взрослых в структуре обращаемости преобладали женщины (83-86%), среди детей наблюдается тенденция к эквивалентной обращаемости мальчиков и девочек (в 2021г – 45% и 55%, соответственно). Медиана возраста взрослых пациентов в 2021г составила 38 лет [31;53], среди детей - 11 лет [7;14]. В 2020г за ТМК обратились жители 74 субъектов РФ, в 2021г – 82 субъектов, при этом отмечается тенденция к преобладанию в структуре ТМК пациентов из Центрального, Приволжского, Южного и Северо-Кавказского федеральных округов. В нозологической структуре ТМК преобладают заболевания щитовидной железы.

**ЗАКЛЮЧЕНИЕ:**

ЗАКЛЮЧЕНИЕ. ТМК оказались востребованы у пациентов с самыми разными эндокринопатиями. Важно проведение дальнейшего анализа как рынка телемедицинских услуг, так и эффективности дистанционного консультирования при различных нозологиях для определения места телемедицины в современной структуре здравоохранения и введения ТМК в систему клинических рекомендаций и программ территориальных фондов ОМС

## Abstract (en)

**BACKGROUND:**

BACKGROUND: The COVID-19 pandemic has accelerated the development of telemedicine technologies. Today there is evidence of the successful use of telemedicine in various fields of health care, in particular in endocrinology. At the same time, there is not enough information for effective integration of telemedicine into the management of patients with various endocrinopathies.

**AIM:**

AIM: The aim of this study is a clinical and demographic assessment of the structure of telemedicine consultations (TMC) conducted at the Endocrinology Research Centre in 2020–2021.

**MATERIALS AND METHODS:**

MATERIALS AND METHODS: A single-stage, single-center retrospective study was conducted. The study included all patients who received at least one TMC at the Endocrinology Research Centre in 2020–2021. Clinical and demographic information was analyzed (gender, age of patients, region of residence, ICD-10 code). All patients signed voluntary informed consent for TMC. The obtained data were processed using the Microsoft Office 2013 software package.

**RESULTS:**

RESULTS: In 2020, 1,548 TMC were held, in 2021 — 4180 TMC. Among adults, women predominated in the structure of referrals (83–86%), among children there is a tendency towards equivalent referrals for boys and girls (in 2021 — 45% and 55%, respectively). The median age of adult patients in 2021 was 38 years [31; 53], among children — 11 years [7; 14]. In 2020, residents of 74 regions of the Russian Federation applied for TMC, in 2021 — of 82 regions. There is a tendency towards the prevalence of patients from the Central, Volga, Southern and North Caucasian federal districts in the TMC structure. Diseases of the thyroid gland predominated in the nosological structure of TMC.

**CONCLUSION:**

CONCLUSION: TMC turned out to be in demand in patients with a wide variety of endocrinopathies. It is important to conduct further analysis of both the TMC market and the effectiveness of remote counseling for various nosologies to determine the place of telemedicine in the modern healthcare structure and to introduce TMK into the system of clinical guidelines and programs of territorial compulsory medical insurance funds.

## ОБОСНОВАНИЕ

В течение 2020 г., прошедшего под эгидой пандемии COVID-19, значительно вырос интерес к телемедицине в широком смысле слова: от возможности «бесконтактного» общения с врачом до использования технологий искусственного интеллекта в описании результатов мультиспиральной компьютерной томографии [1]. Согласно терминологии действующего в Российской Федерации законодательства, под телемедициной понимают возможность оказания медицинской помощи с применением телемедицинских технологий путем проведения консультаций и консилиумов, а также дистанционного медицинского наблюдения за состоянием здоровья пациента [2].

Сама идея использования многочисленных информационных технологий для улучшения организации здравоохранения не нова. Так, первые частные случаи применения радио, телеграфа и телефона в медицинских целях относятся к 1850–1920 гг. Впоследствии в том или ином виде телемедицинские технологии использовались многими странами и отдельными медицинскими учреждениями, однако это не носило систематический характер, также не была в достаточной степени готова техническая и законодательная база [3]. В Российской Федерации однозначная легализация телемедицинских технологий произошла с выходом Приказа Министерства здравоохранения Российской Федерации от 30 ноября 2017 г. N 965н, в котором сформулирован Порядок организации и оказания медицинской помощи с применением телемедицинских технологий [4]. С этого момента отмечается неуклонный рост объема рынка телемедицины в России: так, в 2017 г. он составил 2677,17 млн руб., в 2018 г. — 3735,0 млн руб., а в 2019 г. — 4399,0 млн руб. [5].

В 2020 г. в связи с пандемией COVID-19 отмечалось снижение доступности плановой медицинской помощи для населения во всем мире. В Российской Федерации схожие проблемы наблюдались и вне пандемии: так, согласно данным Федеральной службы государственной статистики РФ, в 2016 г. проблему недоступности медицинских услуг отмечал каждый четвертый респондент [6]. В связи с этим закономерно был отмечен и рост интереса к телемедицине как со стороны профессионального сообщества, так и со стороны пациентов [7].

Согласно Федеральному закону № 323 [8], оплачивать телемедицинские консультации (ТМК) можно из всех источников: ОМС, ДМС, личных средств граждан. Многие клиники предлагают ТМК в рамках платных медицинских услуг; в то же время телемедицина в рамках программы государственных гарантий реализована далеко не во всех регионах. Несмотря на успешное функционирование компонента «Телемедицинские консультации» подсистемы Федеральной электронной регистратуры Единой государственной информационной системы в сфере здравоохранения, обеспечивающего проведение консультаций «врач-врач» между специалистами из регионов и врачами федеральных центров, наиболее востребованный сегмент — консультации «врач-пациент» — остается нереализованным. В некоторых регионах тарифы на онлайн-прием врача-специалиста разработаны (например, согласно тарифным соглашениям ТФОМС в 2020 г. в Брянской области ТМК стоила 406,43 руб. [9]), однако не уточняется, что именно входит в понятие ТМК. В других регионах тарифы не разработаны: например, в Москве, несмотря на активную работу телемедицинских центров по наблюдению за пациентами с COVID-19, ТМК не были внесены в тариф ОМС на 2021 г. [10].

В то же время телемедицинские технологии являются уникальным инструментом, позволяющим пациентам с хроническими заболеваниями систематически наблюдаться у лечащего врача. Так, применение ТМК достоверно улучшает контроль гликемии по сравнению с традиционными консультациями [11], в частности, у детей [12] и пожилых [13]; позволяет достичь большего снижения веса у пациентов с ожирением [14]. Обсуждаются перспективы телемедицины в аспекте различных заболеваний щитовидной железы [15]. Однако на сегодняшний день информации, которая бы позволила эффективно интегрировать телемедицину в ведение пациентов с различными эндокринопатиями, недостаточно. Отсутствует информация об оптимальном формате и частоте ТМК, эффективности дистанционных образовательных программ для пациентов. Исследования, касающиеся удовлетворенности пациентов таким видом консультаций, также сильно лимитированы [16].

В связи с этим ценным представляется опыт ФГБУ «НМИЦ эндокринологии» Минздрава России, экспертного учреждения в области эндокринопатий и ассоциированных с ними состояний.

## ЦЕЛЬ ИССЛЕДОВАНИЯ

Целью данной работы является клинико-демографическая оценка структуры ТМК, проведенных в ФГБУ «НМИЦ эндокринологии» в 2020–2021 гг.

## МАТЕРИАЛЫ И МЕТОДЫ

Место и время проведения исследования

Место проведения. ФГБУ «НМИЦ эндокринологии» Минздрава России.

Время исследования. Январь 2020 г. – декабрь 2021 г.

Изучаемые популяции

В исследование были включены все пациенты, самостоятельно или посредством родителей/законных представителей подписавшие добровольное информированное согласие на проведение ТМК специалистами ФГБУ «НМИЦ эндокринологии» Минздрава России и получившие хотя бы 1 ТМК за указанный период времени.

Дизайн исследования не подразумевает наличие критериев исключения.

Способ формирования выборки из изучаемой популяции

Применялся сплошной способ формирования выборки.

Дизайн исследования

Проведено одноцентровое обсервационное одномоментное одновыборочное исследование.

Описание медицинского вмешательства

ТМК проводились согласно действующему Приказу Министерства здравоохранения Российской Федерации от 30 ноября 2017 г. N 965н («Порядок организации и оказания медицинской помощи с применением телемедицинских технологий») и СОП «Проведение телемедицинской консультации».

В ФГБУ «НМИЦ эндокринологии» Минздрава России ТМК «врач-пациент» проводятся посредством медицинской информационной системы qMS, интегрированной с Личным кабинетом пациента на сайте Центра. Идентификация и аутентификация пациента производятся за счет интеграции Личного кабинета с порталом Госуслуг (т.е. при помощи простой электронной цифровой подписи, ЭЦП). Врач подписывает электронный документ (консультацию) при помощи квалифицированной ЭЦП.

Согласно Порядку оказания медицинской помощи с применением телемедицинских технологий [4], в Центре осуществляется два вида ТМК. Первые, заочные, доступны для пациентов, которые ранее не были на приеме у специалиста Центра и проводятся по документам. Результатом консультации являются рекомендации по необходимому дообследованию, очной консультации специалиста. Данный вид ТМК в 2020 г. был запущен в пилотном режиме для врачей-аритмологов и радиологов.

Второй тип консультаций доступен для пациентов, которые ранее уже были на очном приеме у любого из специалистов Центра. В этом случае лечащий врач может уточнить диагноз, скорректировать медикаментозную терапию. Консультации с лечащим врачом проводятся в двух форматах — с видеосвязью и без видеосвязи.

ТМК «врач-пациент», с учетом отсутствия соответствующих услуг и тарифов в программах ТФОМС, проводятся на основе договора об оказании платных медицинских услуг или же за счет иных источников финансирования (например, грантов). ТМК «врач-врач», проводящиеся специалистами Центра через систему Всероссийского центра медицины катастроф «Защита» Федерального медико-биологического агентства, в представленные расчеты не включались.

Методы

Анализировалась клинико-демографическая информация о проведенных ТМК (пол, возраст пациентов, регион проживания, код заболевания по МКБ-10). Данные нозологической структуры обращаемости анализировались только за 2021 г., поскольку 2020 г., ввиду пандемии и относительно низкой осведомленности больных о возможностях дистанционного консультирования, расценен нами как недостаточно репрезентативный.

Статистический анализ

Анализ полученных данных проводился при помощи пакета программ Microsoft Office версии 2013 г. Данные представлены в виде медиан [Q1; Q3], если не указано иное.

Этическая экспертиза

Протокол исследования одобрен локальным Этическим комитетом ФГБУ «НМИЦ эндокринологии» Минздрава России. Было постановлено одобрить возможность проведения одноцентрового обсервационного одномоментного одновыборочного неконтролируемого исследования, посвященного клинико-демографическому анализу телемедицинских технологий, проведенных в ФГБУ «НМИЦ эндокринологии». Научная работа соответствует этическим стандартам добросовестной клинической практики.

## РЕЗУЛЬТАТЫ

Общая характеристика амбулаторной обращаемости в НМИЦ эндокринологии

Всего в Центре в 2020 г. было проведено 51 967 амбулаторных консультаций (учитывались консультации, проведенные по договору, в рамках грантовой поддержки или в счет ОМС). 44 426 (77%) консультаций было проведено взрослым пациентам, 7541 (23%) — детям. В 2021 г. амбулаторно было проведено 75 522 консультации, из них 62 451 (83%) консультация — взрослым, 13 071 (17%) — детям.

В 2020 г. соотношение мужчин и женщин составило 10 238:34 188 (т.е. 23,1% и 76,9% соответственно). Медиана возраста всех взрослых пациентов составила 45 лет [ 33; 58]. Среди детей 4401 консультация была проведена девочкам, 3140 — мальчикам (т.е. 58,4 и 41,6% соответственно). Медиана возраста детей составила 11 лет [ 7; 14].

В 2021 г. соотношение мужчин и женщин составило 14 433:48 013 (т.е. 36,4 и 63,6% соответственно). Медиана возраста всех взрослых пациентов составила 45 лет [ 33; 59]. Среди детей 7479 консультаций было проведено девочкам, 5592 — мальчикам (т.е. 58,4 и 41,6% соответственно). Медиана возраста детей составила 12 лет [ 8; 15].

Нозологическая структура обращаемости за амбулаторными консультациями представлена на рис. 1.

Первые «пилотные» консультации «врач-пациент» были проведены в НМИЦ эндокринологии в 2019 г. (тогда суммарно было проведено 104 консультации). Далее, за 2020 г., в Центре было проведено 1548 консультаций «врач-пациент» (т.е. практически в 15 раз больше, чем в 2019 г.). В 2021-м обращаемость за ТМК в нашем центре также возрастала: было проведено 4180 консультаций (т.е. на 270% больше, чем в 2020 г.). Сравнительная динамика количества ТМК представлена на рис. 2.

**Figure:**
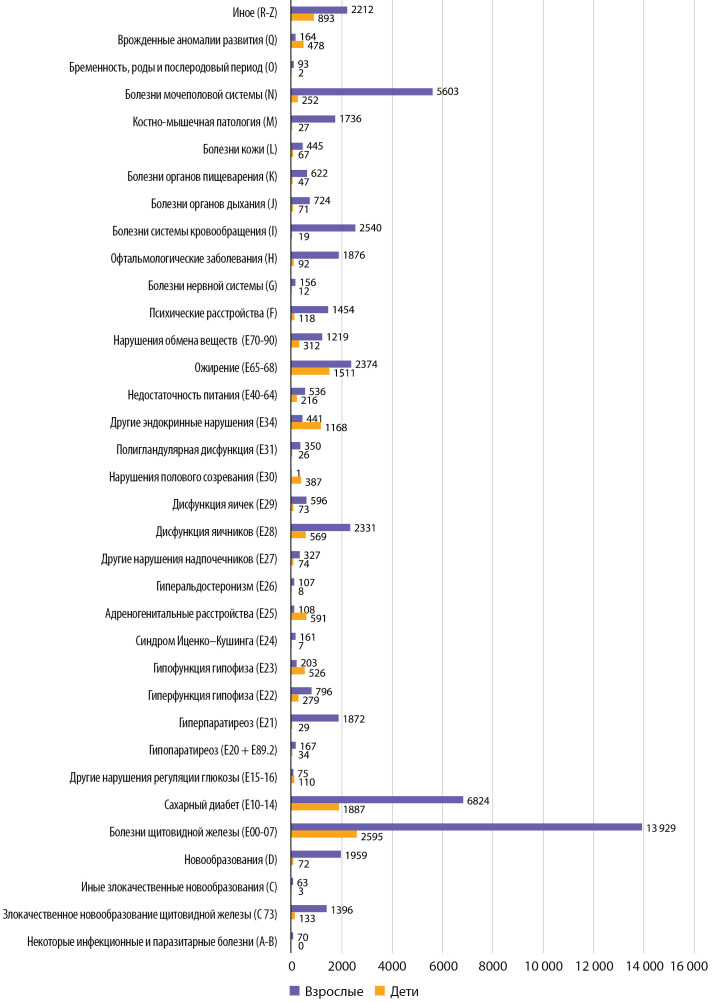
Рисунок 1. Обращаемость за очными амбулаторными консультациями в 2021 г. По оси абсцисс — абсолютное число консультаций, по оси ординат — нозология согласно МКБ-10.

**Figure:**
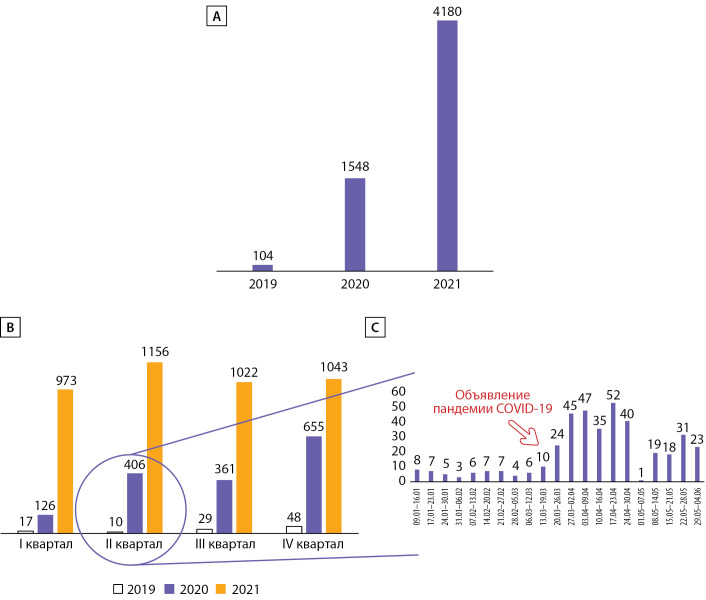
Рисунок 2. А — сравнительная динамика количества телемедицинских консультаций в 2019–2021 гг. В — то же с детализацией по кварталам. С — увеличение востребованности ТМК после объявления пандемии новой коронавирусной инфекции в 2019 г.

Среди всех консультаций, проведенных пациентам старше 18 лет, с видеосвязью в 2021 г. было проведено 1032 ТМК (25% всех ТМК), для сравнения — в 2020 г. с видеосвязью было проведено 566 ТМК (36,5%). Распределение ТМК с видеосвязью сохраняется стабильным: 19% таких консультаций проведено для пациентов младше 18 лет, 81% — для взрослых пациентов. Далее приведена детальная характеристика ТМК, проведенных в НМИЦ эндокринологии в 2021 г.

Демографическая характеристика обращаемости за ТМК

Взрослым пациентам (в возрасте 18 лет и старше) в 2020 г. было проведено 1200 ТМК, в 2021 г. — 2842 ТМК, при этом в 2021 г. 83% ТМК было оказано женщинам, 17% — мужчинам. Указанная гендерная асимметрия носит стойкий характер: в 2020 г. на женщин пришлось 86% ТМК, на мужчин — 14%.

При исключении из анализа повторных ТМК установлено, что за телемедицинской помощью в 2020 г. в центр обратились 930 взрослых пациентов, в 2021 г. — 2073 пациентов. В 2020 г. медиана возраста всех взрослых пациентов составила 38 лет [ 31; 53], среди женщин — 37,5 года [ 30; 52], среди мужчин — 42,5 года [ 34,25; 54]. В 2021 г. медиана возраста всех взрослых пациентов составила 39 лет [ 31; 51], среди женщин — 39 лет [ 31; 51], среди мужчин — 40 лет [ 31,75; 51]. Таким образом, ТМК остаются наиболее востребованы именно среди пациентов средней возрастной категории. Распределение пациентов по полу и возрасту представлено на рис. 3.

Детям в 2021 г. было проведено 1338 ТМК, в 2020 г. — 348 ТМК. Интересно, что в 2020 г. на долю мальчиков пришлось 27% ТМК, а в 2021 г. — 45%.

При исключении из анализа повторных ТМК установлено, что в 2020 г. ТМК были оказаны 272 детям, в 2021 г. — 664 детям. В 2020 г. медиана возраста детей составила 10 лет [ 6; 14], среди девочек — 11 лет [ 6,25; 14], среди мальчиков — 10 лет [ 4; 13]. В 2021 г. медиана возраста детей составила 11 лет [ 7; 14], среди девочек — 11 лет [ 7; 14], среди мальчиков — 11 лет [ 7; 14]. Графически распределение детей по полу и возрасту представлено на рис. 4.

**Figure:**
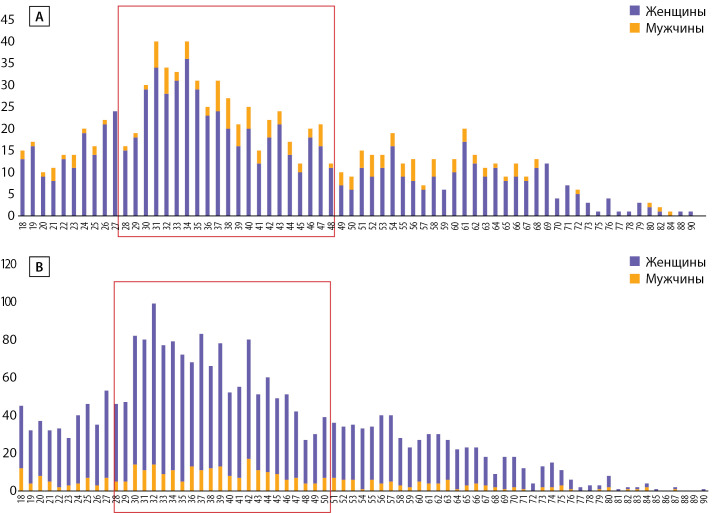
Рисунок 3. А, В — количество пациентов старше 18 лет в зависимости от возраста (А — 2020 г., В — 2021 г.). По оси ординат отмечено абсолютное количество ТМК, по оси абсцисс — возраст пациентов. Красным отмечены наиболее активно пользующиеся ТМК возрастные категории.

**Figure:**
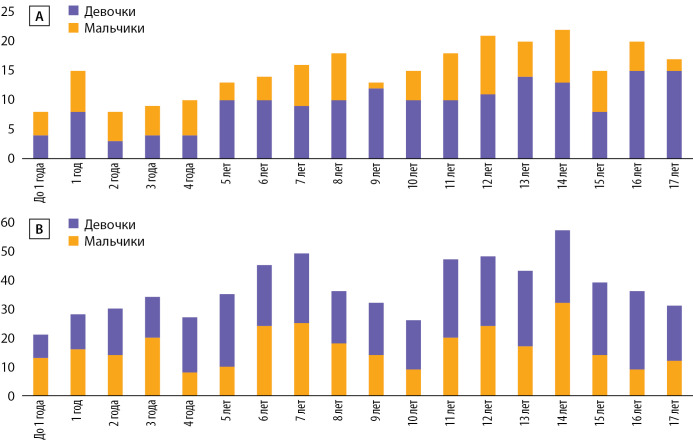
Рисунок 4. А, В — количество пациентов младше 18 лет в зависимости от возраста (А — 2020 г., В — 2021 г.).По оси ординат отмечено абсолютное количество ТМК, по оси абсцисс — возраст пациентов.

Территориальная структура обращаемости за ТМК

В 2020 г. за ТМК в НМИЦ эндокринологии обратились жители 74 субъектов РФ. Медиана количества ТМК на каждый субъект составила 5 [ 3; 9] консультаций. В 2021 г. за ТМК в НМИЦ эндокринологии обратились жители 82 субъектов РФ. Медиана количества ТМК на каждый субъект составила 13 [ 8; 25] консультаций. На рис. 5 представлено распределение количества ТМК по субъектам, на рис. 6 эти данные представлены в виде карты.

Нозологическая структура обращаемости за ТМК

В 2021 г. все ТМК были проведены по 273 нозологиям (в соответствии с кодами по МКБ-10). Перечень кодов МКБ-10 согласно рубрикатору представлен на рис. 7.

Для отдельного анализа мы выделили нозологии, по поводу которых взрослым было проведено более 50 ТМК, а детям — более 20 ТМК за 2021 г. Данные представлены на рис. 8.

**Figure:**
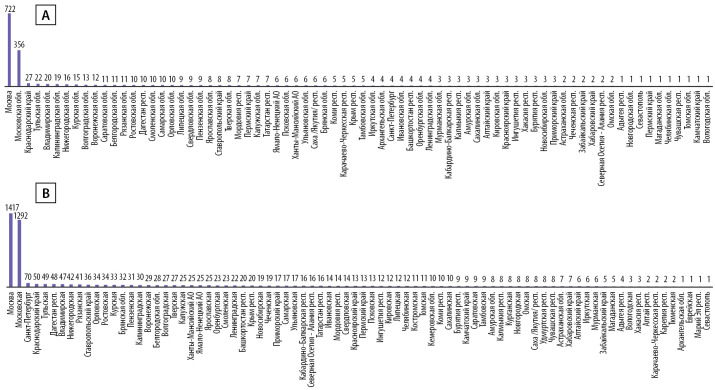
Рисунок 5. Распределение ТМК по субъектам РФ: А — 2020 г.; В — 2021 г. По оси абсцисс отмечено количество ТМК.

**Figure:**
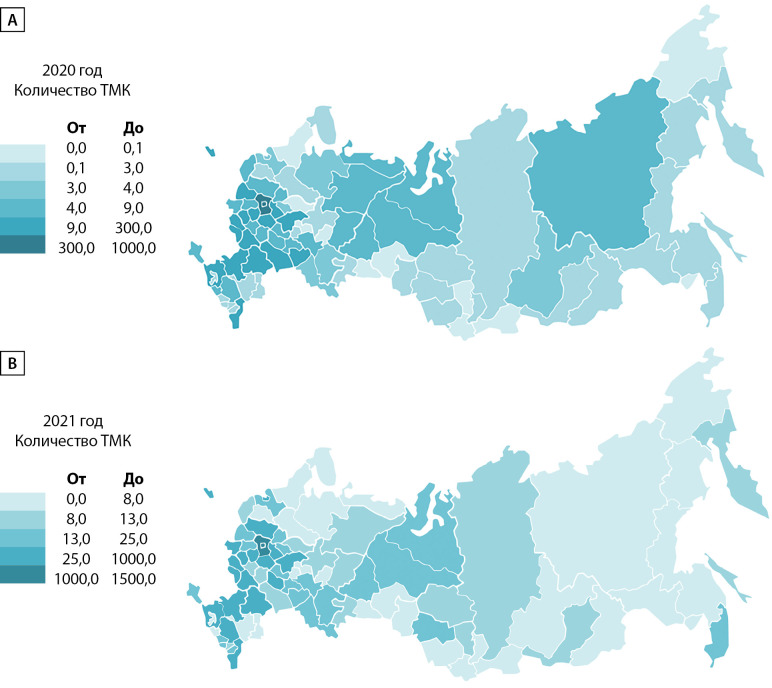
Рисунок 6. Графическое отражение распределения количества ТМК по субъектам РФ в виде карты федеративного устройства РФ: А — 2020 г.; В — 2021 г., цветовые легенды представлены рядом с картами (более темный оттенок означает большее количество ТМК).

**Figure:**
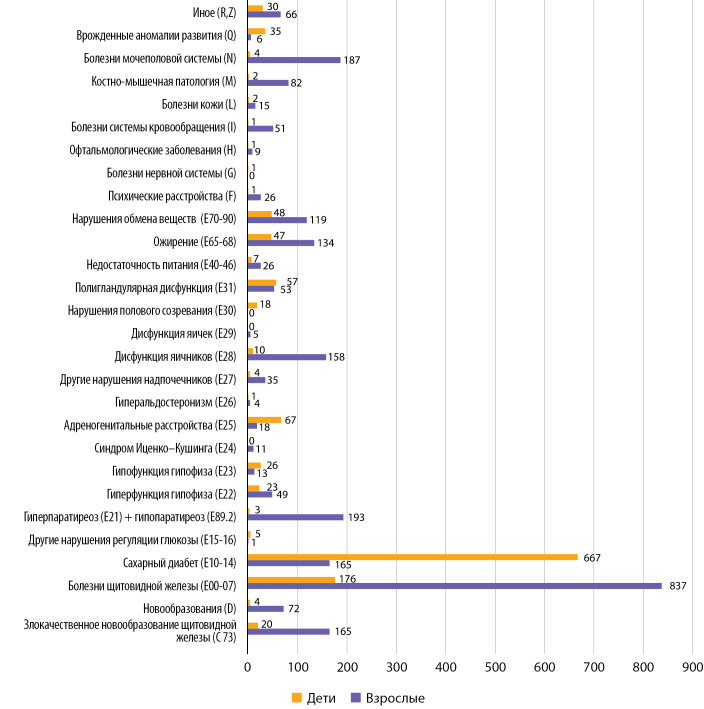
Рисунок 7. Перечень нозологий, с которыми пациенты обращались за ТМК в 2021 г. По оси абсцисс отмечено абсолютное число ТМК по той или иной группе нозологий.

**Figure:**
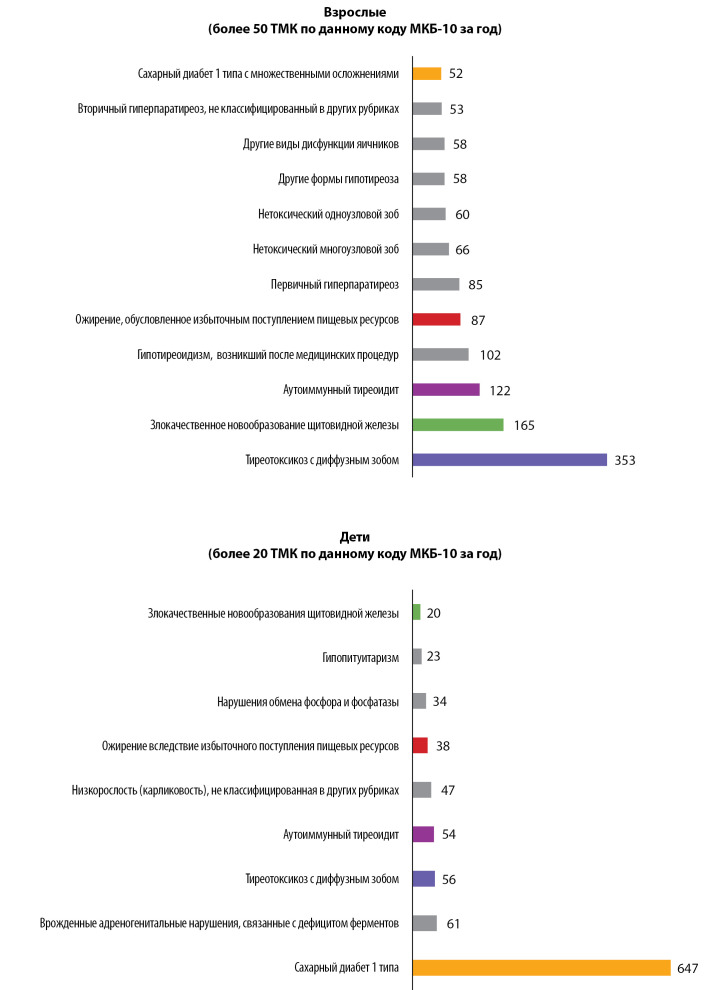
Рисунок 8. Перечень нозологий, с которыми пациенты наиболее часто обращались за ТМК в 2021 г. По оси абсцисс отмечено абсолютное число ТМК по той или иной группе нозологий.

## ОБСУЖДЕНИЕ

Репрезентативность выборок

Набор участников проводился только в ФГБУ «НМИЦ эндокринологии» Минздрава России, что ограничивает репрезентативность полученной выборки.

Сопоставление с другими публикациями

После объявления ВОЗ в марте 2020 г. пандемии новой коронавирусной инфекции был отмечен резкий скачок востребованности дистанционных консультаций, что согласуется с мировыми данными [17]. Наблюдаемый спрос не уменьшился со спадом заболеваемости летом 2020 г. Также в марте и апреле 2020 г. было проведено интенсивное обучение всех врачей-специалистов проведению ТМК, в срочном порядке были изготовлены ЭЦП, проведено информирование пациентов о возможности ТМК средствами смс-оповещений, информации на сайте Центра, были разработаны специальные видеоинструкции и памятки. Благодаря совокупности этих факторов был достигнут высокий темп роста востребованности ТМК, сохранившийся и к концу 2021 г.

Обращает на себя внимание значительное преобладание женщин в структуре обращаемости за ТМК. Это соответствует международным данным о том, что женщины в принципе более часто обращаются за медицинской помощью [18–20], а также соответствует общей амбулаторной обращаемости в Центр. При этом в отношении детей (на консультацию с которыми обращаются их законные представители, родители) соотношение мальчиков и девочек выравнивается.

Наиболее активными пользователями ТМК оказались пациенты среднего возраста. Это объясняется, с одной стороны, в целом более удовлетворительным состоянием здоровья молодой категории населения [21], а с другой — более низкой доступностью информационных технологий для пожилых [22].

Анализ территориальной структуры обращаемости за ТМК представляет несколько парадоксальные данные: несмотря на близость Центра к пациентам г. Москвы и Московской области, жители именно этих субъектов чаще всего обращались за ТМК в 2021 г. При этом в отношении остальных субъектов зачастую количество ТМК тем больше, чем ближе субъект находится к Москве. У этого феномена может быть несколько причин.

Во-первых, Центр преимущественно проводит повторные ТМК (т.е. на дистанционные консультации записываются те пациенты, которые уже были в Центре очно). Естественно, что по территориальному признаку первично очно обратиться в Центр удобнее именно жителям Москвы и Московской области. С другой стороны, на стационарное обследование и лечение в Центр поступают большое количество пациентов из различных субъектов РФ.

Во-вторых, согласно действующему законодательству [4], при первичном дистанционном приеме врач вправе только назначить обследование, но не может ни назначить лечение, ни поставить диагноз. В связи с этим пациенты могут либо разочаровываться в первичных дистанционных консультациях, либо сознательно отказываться от таковых в пользу полноценных очных визитов.

В-третьих, в удаленных регионах Российской Федерации, по данным Росстата [23], ниже доступность как персональных компьютеров, так и доступа в Интернет, что также ограничивает возможности пациентов в отношении ТМК.

И, наконец, важную роль может играть и наличие в самом субъекте либо недалеко от него профильного учреждения с достаточным спектром компетенций в конкретной области (напр., НМИЦ им. В.А. Алмазова в Северо-Западном федеральном округе).

Наибольший практический интерес представляет нозологическая структура обращаемости за ТМК. Среди патологий, с которыми взрослые пациенты обратились за ТМК наибольшее количество раз (см. рис. 8), с огромным отрывом лидируют заболевания щитовидной железы, патологии минерального обмена, различные заболевания яичников. Это не согласуется с эпидемиологическими данными о распространенности данных эндокринопатий и с общей структурой амбулаторной обращаемости в Центр, в особенности в отношении сахарного диабета.

Такая сравнительно низкая обращаемость среди пациентов с сахарным диабетом (особенно среди пациентов с сахарным диабетом 2 типа), вероятнее всего, является результатом реализации подпрограммы «Сахарный диабет» федеральной целевой программы «Предупреждение и борьба с социально значимыми заболеваниями» в 2007–2012 гг.. Именно тогда в Российской Федерации была на высоком уровне организована диабетологическая помощь; результатом реализации программы стало значимое увеличение продолжительности и качества жизни пациентов [24]. Вероятно, «отголоски» реализации программы сейчас дают относительно высокую доступность помощи при сахарном диабете по сравнению с иными эндокринопатиями. В связи с этим пациентам может быть удобнее наблюдаться в медицинских организациях по месту жительства, а не обращаться за помощью в федеральные учреждения на регулярной основе.

Однако свой вклад могут вносить и более тонкие особенности. Так, возможно, что пациентам с сахарным диабетом неудобен формат разовых консультаций, и была бы востребована возможность чата с лечащим врачом для решения текущих вопросов (например, при подборе доз инсулинотерапии). Кроме того, перспективным представляется создание приложения, интегрирующего возможности дневника самоконтроля с «супервизией» лечащего врача [25]. Необходимо отметить, что такого рода помощь попадает под понятие дистанционного мониторинга, использование которого, согласно действующему законодательству, сопряжено с рядом технических трудностей, в свою очередь делающих такой вид ТМК по сути неприменимым. Таким образом, для более эффективного использования ТМК в отношении пациентов с сахарным диабетом может требоваться пересмотр и правовой базы.

С другой стороны, пациенты с сахарным диабетом 2 типа характеризуются относительно низким комплаенсом, и низкая приверженность динамическому наблюдению может распространяться и на телемедицинские технологии [26].

По всей видимости, важен и вопрос финансирования ТМК: относительно большое число ТМК по поводу сахарного диабета 1 типа у детей мы связываем с наличием грантовой поддержки этого вида ТМК, что делает указанные консультации для пациентов бесплатными. К сожалению, аналогичные программы по поддержке взрослых больных сахарным диабетом (как 1, так и 2, и других типов) крайне лимитированы. Необходимо детальное изучение ключевых звеньев, на которых ТМК могут повлиять на приверженность пациентов к лечению и, соответственно, профилактировать развитие осложнений и смертность.

Клиническая значимость результатов

Согласно нашим данным, для определенных групп пациентов (например, взрослых пациентов с диффузным токсическим зобом или детей с адреногенитальными нарушениями) ТМК представляются удобным и востребованным способом контакта с лечащим врачом.

Ограничения исследования

Проведенное исследование является пилотным, описательным и имеет ряд ограничений. Так, не проводилось сравнение эффективности и безопасности дистанционного и очного амбулаторного наблюдения пациентов с различными эндокринопатиями. Выборка ограничена пациентами ФГБУ «НМИЦ эндокринологии», учреждения третьего уровня, куда чаще обращаются пациенты с тяжелыми и нетипичными формами заболеваний.

Направления дальнейших исследований

ТМК, по всей видимости, представляются удобным инструментом в наблюдении пациентов с рядом нозологий. Необходимо проведение проспективных исследований, посвященных оценке эффективности и безопасности ТМК при различных эндокринных заболеваниях. Также необходим клинико-экономический анализ применения ТМК для таких «востребованных» нозологических групп с рассмотрением вопроса о внедрении ТМК в стандарты медицинской помощи при соответствующих заболеваниях.

## ЗАКЛЮЧЕНИЕ

В последние годы телемедицинские технологии активно развиваются как за счет совершенствования технологий и нормативно-правовой базы, так и в связи с повышением спроса на дистанционные консультации ввиду распространения новой коронавирусной инфекции. ТМК оказались востребованы у пациентов с самыми разными эндокринопатиями. Важно проведение дальнейшего анализа как рынка ТМК, так и эффективности дистанционного консультирования при различных нозологиях для определения места телемедицины в современной структуре здравоохранения и введения ТМК в систему клинических рекомендаций и программ территориальных фондов ОМС.

## ДОПОЛНИТЕЛЬНАЯ ИНФОРМАЦИЯ

Источники финансирования. Работа выполнена по инициативе авторов без привлечения финансирования.

Конфликт интересов. Авторы декларируют отсутствие явных и потенциальных конфликтов интересов, связанных с содержанием настоящей статьи.

Участие авторов. Горбачева А.М. — концепция и дизайн исследования, получение, анализ данных, интерпретация результатов, написание рукописи; Логвинова О.В. — концепция и дизайн исследования, написание статьи; Мокрышева Н.Г. — концепция и дизайн исследования, внесение в рукопись существенных правок с целью повышения научной ценности статьи. Все авторы одобрили финальную версию статьи перед публикацией, выразили согласие нести ответственность за все аспекты работы, подразумевающую надлежащее изучение и решение вопросов, связанных с точностью или добросовестностью любой части работы.
